# The Unique Challenges and Contributions of Diverse Caregivers

**DOI:** 10.1093/geroni/igaa057.2373

**Published:** 2020-12-16

**Authors:** Rita Choula

**Affiliations:** AARP, Washington, District of Columbia, United States

## Abstract

Caregiving in the U.S. 2020 oversampled African Americans, Hispanics, Asians, and people over the age of 75. Six in ten caregivers report being non-Hispanic white, 17% are Hispanic, 14% non-Hispanic African-American or black, 5% Asian/Pacific Islander, and 3% some other race or ethnicity, including multiracial. The session will emphasize the unique context of diverse caregivers, including African American, Hispanic, Asian, and LGBT+ caregivers. The session will begin by discussing the portrait of the typical caregiver of each of these groups. It will follow with a discussion of the challenges facing diverse caregivers in the aggregate and the opportunities to recognize and support them across settings.

